# The Conduct of Competitions: A Suggestion

**Published:** 1912-07-20

**Authors:** 


### HOSPITAL ARCHITECTURE AND CONSTRUCTION.

[Communications on this subject should be marked "Architecture" in the left-hand top corner of the envelope.]

### The Conduct of Competitions: A Suggestion.

The opinion expressed on page 563 of our issue of March 2, that no adequate system exists in this country to ensure that hospital authorities by architectural competition get what they require, seems to have drawn from fairly representative members of the profession a concurrence with our repeatedly expressed statements. Architects are somewhat protected by their Royal Institute in matters relative to open competitions by regulations recently revised; and when competitions are mooted not in accordance with these regulations the members and licentiates are not permitted to compete. Six regulations are described as essentials: the first is, "There shall be appointed one or more fully qualified professional assessors, to whom the whole of the designs shall be submitted." It is further suggested that the selection of these assessors should be made with the greatest possible care, as the successful result of the competition will depend very largely on his or their experience and ability.

The regulations go on to describe three different manners in which competitions may be conducted. The first method applies to competitions for public works of great architectural importance, and the procedure is to advertise in the Press, inviting architects willing to compete to send in designs according to certain obtainable conditions and requirements. We would suggest that in competitions of this nature the Royal Institute should advise that not fewer than two assessors be appointed. Such a course would not be any more costly, for it is laid down in Clause 8: "The scale of charges for assessing competitions, whether by jury or otherwise, is the sum of thirty guineas, plus one-fifth per cent. upon the estimated cost of the proposed building." We have precedent for the suggestion in the case of the competitions for the London County Hall and for the National Museum of Wales; and, further, there are competitions conducted by the Royal Institute itself on such lines for the various prizes and studentships it has the privilege to award. Every competition of magnitude causes heart-burning to some competitors, but few realise the difficulties which hedge around an assessor; if he draws up the conditions and requirements on which the competitors have to work, he cannot help having some slight prejudice as to the general lay-out of the design. Where only one assessor is appointed, members of the profession can play up to him by a diligent study of his completed works; to do so is quite honourable, for the assessor is presumably appointed on the basis of his meritorious works and achieved position. The assessor has to enter on his duties with an absolutely unbiased mind, if such is possible, and he has, further, to reduce all the designs to one level standard of draughtsmanship, which is not so easy as one might think. He has then to eliminate designs which do not conform to the conditions, which is often difficult, for this reason: as a competitor causes his design to grow and become real to him, he may see that by infringing a certain condition, maybe a needless one, he can give an infinitely better result; he does it, and what can the assessor do?

Two courses are open to him: he may disqualify the design, or frankly admit that the competitor has seen farther than he did when he composed the conditions. These few points serve to illustrate that loyalty to an award is, in nine cases out of ten, morally and professionally justified. It must also not be forgotten that architects are professionally engaged in works as far apart as the poles in general character, and they cannot, therefore, as they are human, be conversant with most recent developments in each branch of the very varied types of buildings, etc. Would it not, therefore, be expedient for the Royal Institute to suggest in their regulations for competitions, that where designs for highly complex buildings are required the promoters should consider whether they ought not to appoint a specialist to collaborate with the architect as assessor? Such a course would free the architectural profession from a certain amount of partly justified criticism. In hospital planning, the system of combined assessors has been proved satisfactory.

